# Circumcision as an Intervening Strategy against HIV Acquisition in the Male Genital Tract

**DOI:** 10.3390/pathogens10070806

**Published:** 2021-06-25

**Authors:** Adhikarimayum Lakhikumar Sharma, Joseph Hokello, Mudit Tyagi

**Affiliations:** 1 Center for Translational Medicine, Thomas Jefferson University, 1020 Locust Street, Philadelphia, PA 19107, USA; LakhikumarSharma.Adhikarimayum@jefferson.edu; 2 Department of Basic Science, Faculty of Science and Technology, Kampala International University-Western Campus, Bushenyi P.O. Box 71, Uganda; hokello.joseph@kiu.ac.ug

**Keywords:** HIV, viral transmission, circumcision, heterosexual HIV-1 transmission

## Abstract

Unsafe sex with HIV-infected individuals remains a major route for HIV transmission, and protective strategies, such as the distribution of free condoms and pre-or post-prophylaxis medication, have failed to control the spread of HIV, particularly in resource-limited settings and high HIV prevalence areas. An additional key strategy for HIV prevention is voluntary male circumcision (MC). International health organizations (e.g., the World Health Organization, UNAIDS) have recommended this strategy on a larger scale, however, there is a general lack of public understanding about how MC effectively protects against HIV infection. This review aims to discuss the acquisition of HIV through the male genital tract and explain how and why circumcised men are more protected from HIV infection during sexual activity than uncircumcised men who are at higher risk of HIV acquisition.

## 1. Introduction

Over the past four decades, major global efforts have been initiated to address the HIV/AIDS epidemic, and significant progress has been made in terms of controlling the disease spread and in therapeutics. Current treatment regimens are highly effective at reducing the amount of HIV in the body (viral load) to very low and undetectable levels [1]. However, while current treatment regimens are very effective and have improved the life expectancy of HIV-infected individuals, they fail to eradicate the virus from the body [2,3,4,5,6,7,8,9]. If treatment is halted, the virus re-emerges, even if it has been suppressed for many years. This is because the virus can continue replicating in immune-privileged sites, where the drugs have limited access (for example, the brain), and also because the virus can establish latent infection [10]. Therefore, the infected individuals need to rely on highly active antiretroviral therapy (HAART) for the rest of their life to suppress the viral load in the blood.

To deal with HIV under this current scenario, prevention is the best strategy. Whilst most HIV new infections are due to sexual transmission, prevention strategies like abstinence, using condoms during sex, taking prevention medicines such as pre-exposure prophylaxis and post-exposure prophylaxis are some of the effective options to prevent HIV infection. Nevertheless, these strategies are not always used nor are they always available, and clinicians and policymakers are in search of alternative strategies in resource-limiting settings and high HIV prevalence areas.

Studies (including clinical trials) have been conducted to investigate the effect of male circumcision (MC) on the acquisition of HIV, and findings suggested that circumcised men are at lower risk of acquiring HIV infection during sexual intercourse [11,12,13]. Moreover, MC is also reported to be effective for preventing the spread of sexually transmitted diseases such as gonorrhea and syphilis [14]. In one study, medical MC reduced the risk of acquisition of HIV by heterosexual men by 38% to 66% over 24 months [15]. Another systematic and meta-analysis study in low and middle-income countries showed that circumcision is more likely to protect (23% reduced odds of HIV infection) men who have sex with men from HIV [16]. However, to implement this strategy on a larger scale, a wider understanding is needed regarding the exact mechanism for how MC protects against HIV infection. Thus, based on the literature available to date, this review discusses the acquisition of HIV in the male genital tract and explains how and why circumcised men are well protected from HIV infection during heterosexual activity, unlike uncircumcised men who are at higher risk of HIV acquisition.

## 2. Anatomy of Male Genital Tract and Risk of HIV Infection

Men become infected with HIV mostly when they are involved in sexual interaction with infected individuals through vaginal sex (insertive vaginal sex; 0.04%) [17] and anal sex (insertive anal sex; 0.06–0.62%) [18] via their penis. This indicates that the penis plays an important role in HIV transmission. Therefore, understanding the changes in the anatomy of the penis during and after sexual intercourse becomes important.

### 2.1. Anatomy of the Male Genital Tract

The penis consists of the penile shaft, glans, urethral meatus, the inner and outer foreskin surfaces, the frenulum, and the thin band [19]. The foreskin (prepuce) is the double-layered fold of smooth muscle tissue, neurons, blood vessels, skin, and mucous membrane part of the penis. The prepuce protects the glans penis and the urinary meatus. The outside of the foreskin has a continuation of the skin on the shaft of the penis while the inner foreskin is a mucous membrane. The foreskin is attached to the glans by a frenulum which is a highly vascularized tissue of the penis. The penile shaft and outer foreskin surfaces are keratinized and contain stratified squamous epithelium. In contrast, the inner foreskin is a wet mucosal surface that is rich in Langerhans Cells (LCs). Moreover, the inner mucosal surface of the foreskin is not keratinized and susceptible to abrasions, facilitating the entry of infectious agents.

### 2.2. Exposure of Inner Foreskin and Trapping Infectious Secretion under Foreskin Increase the Risk of Infection

During sexual intercourse, the foreskin of the penis is pulled back down the penile shaft thereby the entire inner foreskin surface becomes exposed to the biological fluids including the infected virginal secretions [19,20]. The inner surface of the human foreskin is highly susceptible to HIV-1 infection [21]. As the inner foreskin is known to be rich in immune cells, the act of sexual intercourse by an uncircumcised man with an infected partner increases the chances of HIV infection by exposing a large surface area where HIV transmission can occur [20].

After sexual intercourse with an infected partner, the penis returns to a flaccid state, in which there is a high chance of trapping the infectious materials (infected vagina secretion) in a moist, warm and favorable environment [20,22]. The inner lining of the prepuce is not keratinized, thin, and easily torn, and has an abundance in HIV target cells. Therefore, this inner lining of the prepuce and prepuce sac provides an extra surface for HIV infection.

### 2.3. Abundance of Immune Cells in the Inner Foreskin Increase the Risk of HIV Infection

A primary target of HIV infection, antigen-presenting cells (APC), are present in the mucosa of the inner foreskin and urethra [19,23]. The penile foreskin has high densities of Langerhans cells (LCs), dendritic cells, and also CD4 + T-cells along, with the co-receptors CCR5 and CXCR4. Macrophages, another type of possible HIV target cells, are also reported to be present, residing below the epithelium. Overall in the adult foreskin, 22.4%, 11.5%, and 2.4% of the total cell population consisting of CD4 + T-cells, Langerhans cells (LCs) (tissue-resident macrophages of the skin), and macrophages, respectively [20]. A study had also reported that the human penis is an efficient mucosal effector site, containing cell subsets that are required to induce and generate specific and effective immune responses against mucosal pathogens [24]. These immune cells of the inner foreskin normally fight against the invading bacteria and pathogen. However, when these cells are exposed to HIV, they provide a gate for HIV entry. Therefore, the inner foreskin is rich with HIV target cells, and it traps infectious materials in the inner lining of the prepuce, factors that increase the risk of HIV acquisition.

## 3. Cell-Free Transmission and Cell-Associated HIV Transmission

HIV transmission is either by cell-free virus particles or cell-cell contacts [25,26,27].

### 3.1. Cell-Free Transmission of HIV-1

**Figure 1:**
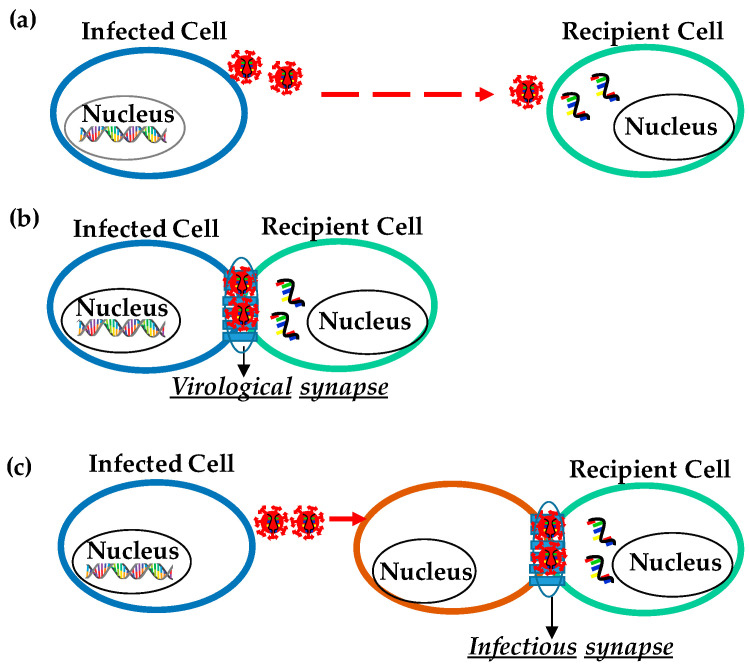
Transmission of HIV. (**a**) Cell-free HIV virion transmission to a distant cell. (**b**) Cell-to-cell transmission through specialized structures called virological synapses. (**c**) Trans-infection, in which virus particles are captured by a cell that itself does not get infected (**orange color**) and then is presented to a target cell (**green color**) at a cell-cell contact designated infectious synapse.

HIV-1 transmission through a cell-free method involves the release of the viral particle from the HIV-1 infected cells, which then infects the uninfected cells at a distance by diffusion through the extracellular space cells (Figure 1a). HIV-1 infects the main subset of T lymphocytes via binding to CD4, the cell receptors expressed by mononuclear phagocytes and dendritic cells. The trimeric HIV-1 envelope glycoprotein (Env) is responsible for the recognition of the cell receptors and its entry into the cytoplasm. Env is expressed as gp160 but proteolytically cleaved into gp120 and gp41 heterodimers. Three heterodimers of gp120 and gp41 are assembled forming the final trimeric Env spike [28]. The Env trimer readily dissociates into gp120 and gp41 subunits that can specifically infect CD4+ cells, which include T helper cells, macrophages, microglial cells, and dendritic cells (DCs). For cellular entry, HIV needs to make contact through its envelope glycoproteins (gp120) with both the receptor CD4 [29] and co-receptor either CCR5 [30] or CXCR4 [31]. Interaction with the appropriate chemokine receptor CCR5 or CXCR4 triggers the conformational changes resulting in the fusion between the viral and cellular membrane. Besides, immune cells, such as Langerhans cells (LCs), dendritic cells (DCs) [23,32,33,34,35] T-cells, and macrophages expressed CD4/CCR5 as principal receptors for HIV-1 [23,33,34,35] and other alternative HIV-1 attachment receptors, such as the Dendritic Cell-Specific Intercellular adhesion molecule-3-Grabbing Non-integrin (DC-SIGN) on DCs and C-type lectins langerin on LCs [34,36,37]. Cell-free HIV transmission allows the spread of the virus over a long distance as they are unrestricted cell-cell contacts and permits an easier spread to a new host [38]. To spread the HIV-1 efficiently through cell-free virus particles: HIV-1 gene expression should be high to assemble and release the virus; cellular factors must be sufficiently expressed to support the assembly and release; HIV-1 should be able to efficiently release into extracellular space, and the extracellular virus should be able to stabilize, bind, and efficiently enter the target cell. Once these factors are fulfilled, the transmission of HIV-1 is mostly carried out by cell-free virus particles. If these factors are not fulfilled, cell-to-cell contact transmission (contact-dependent) is enforced.

### 3.2. Cell-to-Cell HIV Infection

The delivery of HIV-1 particles to adjacent cells for the establishment of infection via cell contacts is defined as cell-to-cell transmission. Cell-cell transmission of the virus promotes the direct transmission of viruses between adjacent cells. The ability of infected donor cells to infect the non-infected cells through cell-cell contact is described by the concept of the virological synapse (VS) while the ability of a non-infected donor cell to capture the virus and transfer it to a permissive target cell is defined as trans-infection [39]. In both these methods of transfer, unlike cell-free HIV-1 transmission, cell-cell contact viral transmission does not allow the virus to go through various virus-limiting steps, such as neutralizing antibodies and complement. The cell-cell transmission does not require any rate-limiting of the virus life cycle, such as virion attachment. Therefore, the direct cell-cell transmission of the virus is more efficient and rapid when compared to cell-free spread [25]. Cell-cell contact promotes or enhances HIV infection. Studies have also shown that HIV spreads more efficiently by utilizing direct cell-cell contact in vitro [27,40,41]. It leads to maybe 100–1000-fold more efficient infection than cell-free viral particles infection [40,42,43,44]. High-multiplicity of infection at the contact site, efficient integration, and accelerated viral gene expression in the target cell are some of the factors that enhance the efficiency of cell-to-cell HIV infection [38,45,46,47]. In addition, unlike cell-free virion transmission, cell-cell transmission could also increase the likelihood of multiple variant transmission. Nevertheless, the cell-cell transmission of HIV is further dependent on the infectivity of donor cells and susceptibility of recipient cells.

#### 3.2.1. HIV Cell-to-Cell Transmission at the Virological Synapse for Cis-Infection

Cell-cell contact among the HIV-infected and uninfected cells enhances infection through specialized structures called virological synapses (VS) (Figure 1b). VS are molecularly organized cellular junctions that form between the infected (“donor”) and uninfected (“target”) cells to allow cell-to-cell viral transmission. VS provides a tight cleft between an HIV-1 infected cell and a target cell that is formed by adhering plasma membranes of the two opposing cells. In cis infection, the formation of VS requires interaction between HIV glycoproteins on the surface of infected cells with the CD4 on target cells. The formation of VS depends on the interaction between the CD4 receptor and HIV envelope glycoprotein (Env) along with other cell adhesion molecules characteristic of immunological synapses, such as intercellular adhesion molecule (ICAM) and lymphocyte function-associated antigen 1 (LFA-1). During the cell-to-cell HIV transmission, HIV-1 Env, Gag, and CD4 were localized at the contact site in an actin-dependent manner [41]. Recruitment and transfer of Gag through the VS have been observed to occur following cell-adhesion capable of forming large platforms of viral assembly referred to as synaptic buttons [48]. The formation of VS is described as a two-part process: an adhesion triggered by Env-CD4 interaction and a stabilized interaction between cell adhesion molecules (ICAM and LFA). After cell-cell adhesion, VS signaling, partly resembles immune signaling through immunological synapses (IS). The tyrosine kinase Zap70 (a protein that is expressed near the surface membrane of lymphocytes) promotes the recruitment of Gag to the site of contact between cells in the VS, but without the involvement of the T cell receptor.

The transmission of HIV-1 through VS was documented for the first time from a dendritic cell to a T cell [49]. VS were later on again reported to form between infected T cells and target CD4 + T cells [41]. Interactions between infected and uninfected T cells enhance HIV infection as the virus can escape neutralizing antibodies. It has been studied in vitro that HIV is transferred between the infected and uninfected immortalized CD4 + T cell lines; between infected CD4 + T cells and epithelial cells; between virus-pulsed dendritic cells and CD4 + T cells, and between infected macrophages and epithelial cells and CD4 + T cells.

#### 3.2.2. Virus Transmission through Trans-Infection

The major pathogenic process of HIV-1 is the capture of HIV-1 by APCs such as myeloid DCs, macrophages [49,50,51,52], and B lymphocytes [53,54] and then the transfer of HIV-1 to CD4+ T cells, resulting in high levels of virus replication in the T cells [55]. In the case of trans-infection, viruses are captured by a cell without infecting itself and then presented to a target cell through infectious synapse (Figure 1c). Soon after the initiation of cell-cell contact, cellular receptor and cell adhesion molecules are accumulated at the contact site to form a long-lasting contact for viral transfer. It was also reported that monocyte-derived DCs (MDDCs) bind HIV particles in vitro and subsequently form infectious synapses with virus receptor-expressing T cells. The outcome of the APC-to-T cell trans infection process has been considered to be central to the sexual transmission of HIV-1 at mucosal (anal and vaginal) and epidermal (foreskin) sites. Studies have suggested that this type of infection yields a high level of virus replication [56], and the viral production in extracellular fluids from the target cells is much higher than that resulting from HIV-1 cis-infection [46].

## 4. Acquisition of HIV in the Male Genital Tract

The main problem in studying the acquisition of HIV in the male genital tract is the lack of proper in-vitro viral transmission model systems that reflect the complex in-vivo structure of such epithelia [34,57]. However, Dinh et al. explored possible sites of HIV-1 transmission in the penis by observing the interaction of HIV-1 particles with the adult male foreskin and penile tissues from men and rhesus macaques [58]. To better understand the kinetics and dynamics of HIV-1 transmission in men during heterosexual intercourse, using epifluorescent microscopy, Dinh et al. visualized HIV-1 particles entering the penile skin to depths where CD4 + T-cells are found. Moreover, a relatively large number of HIV-1 particles were observed in and around the inner foreskin compared to the outer foreskin. Furthermore, they visualized more HIV-1 particles entering the glans compared to the foreskin tissues. These observations in foreskin tissue explants correlated with the observations in the rhesus macaques. These observations suggest that the foreskin holds the HIV-1 particles much longer, which accordingly, continues to infect the glans penis over time. Although much attention has been focused on the foreskin as the main site for HIV transmission, the penile shaft, glans, and urethra are all potential sites of HIV-1 entry and transmission to the penis during heterosexual intercourse. However, the exact mechanisms for how viruses cross the primary barrier of genital epithelial cells (GECs) remain unclear [59].

To describe the events of HIV-1 entry at the foreskin, Ganor et al. developed models of the adult human foreskin epithelium [34,57]. Using the model, they explained the early step involved in HIV entry in the male foreskin. The study has shown that the efficiency of HIV-1 transmission is more in the inner foreskin than in the outer foreskin. The infectious material is unable to penetrate the outer foreskin due to keratinization, which provides a mechanical barrier against HIV [34]. Using confocal and fluorescence microscopy, they showed that HIV particles remain trapped within the apical keratin layer of the outer foreskin but able to penetrate the epidermal part of the inner foreskin [34].

**Figure 2:**
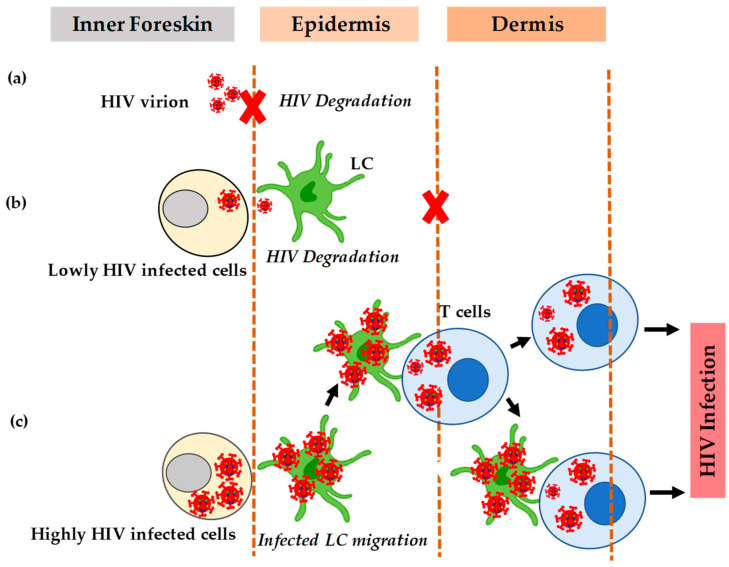
HIV transmission in the inner foreskin. (**a**) The inability of cell-free HIV to translocate efficiently most of it becomes degraded. (**b**) The inefficient translocation of HIV. (**c**) The efficient translocation of HIV when there is a formation of apical viral synapses between cells highly infected with HIV and dendrites of Langerhans cells.

The ability of HIV-infected cells to form VS with apical foreskin keratinocytes makes the HIV-1 translocation more potent. In contrast, cell-free virion does not translocate efficiently, and most of it becomes degraded when taken up by the cells (Figure 2a). Therefore, the efficiency of HIV transmission in the male genital tract also depends on the type of HIV-1 transmission. Since most of the HIV infection in the male genital tract is through cell-to-cell infection, the efficiency of transmission is high. In addition, the translocation of HIV depends primarily on the viral load of the infected partner and the susceptibility of the uninfected counterpart (Figure 2b) [60].

HIV induces chemokines CCL5/RANTES but decreases the secretion of CCL20/MIP-3-alpha that facilitates the redistribution of LC [61]. The increased secretion of CCL5/RANTES also enhances the recruitment of T cells from the dermis to the epidermis [61]. Studies have shown that treatment with tumor necrosis factor-alpha in the inner foreskin of the penis can also activate LC and stimulate cytokines that cause an influx of CD4 + T-cells into the epithelial layer [62,63]. The newly recruited T cells form a conjugate with LC that then transfers HIV-1 to other T-cells. Besides some external factors, the interaction of HIV with the target cells causes the high permeability of HIV in the inner foreskin. Although HIV can infect T cells without LC, the establishment of infection depends on its interaction with LC. Therefore, when exposed to HIV, epidermal Langerhans cells migrate towards the apical surface, internalize HIV, and then transfer it to T-cells (Figure 2c).

Studies have also shown that seminal plasma (SP) induces epithelial cells in the female genitals to secrete chemokines that increase susceptibility to HIV-1 infection. SP may modulate HIV transmission by either enhancing or inhibiting HIV infection and the transfer of viruses [64,65]. Cervicovaginal secretion (CVS) contains a plethora of protective innate factors against HIV-1 [57,66,67,68], indicating that genital fluids may directly modulate HIV-1 transmission. Later on, either SP or CVS alone does not affect HIV-1 but the mixture of SP and CVS does decrease HIV-1 entry in the foreskin, according to both in vitro and in vivo studies [34]. Evidence has shown that STD in uninfected individuals have a higher risk of getting HIV infection than individuals without any STDs [69,70]. It is worth mentioning here that *Chlamydia trachomatis* and *Neisseria gonorrhoeae* are some common STD human pathogens that infect the urethra in men and the cervix in women causing mucosal inflammation [71,72,73]. These pathogens can invade their target epithelial cell that results in the production of proinflammatory cytokines by infected cells and then the influx of various immune cells to the infection site. This process increases the amount of potential HIV-1 target cells thereby enhancing the risk of HIV entry.

## 5. Male Circumcision (MC) and HIV Infection

Based on the biological explanation of HIV entry into the inner foreskin of the penis, the surgical removal of the foreskin of the penis (MC) should reduce the risk of men becoming infected with HIV. Therefore, various studies have been conducted relating to the acquisition of HIV infection in circumcised males compared with uncircumcised males.

### 5.1. Randomized Clinical Trials (RCTs)

Randomized controlled trials (RCTs) have been carried out to determine the effect of MC on HIV acquisition. One RCT recruited 3274 uncircumcised young (18–24 years old) men in the semi-urban region of Johannesburg, South Africa., and then followed them for up to 3, 12, and 21 months [11]. MC was offered to the intervention group immediately after randomization and to the control group at the end of the follow-up [11]. The study showed that MC protected against HIV acquisitions, equivalent to what a vaccine of high efficacy would have achieved [11]. This trial concluded that MC may provide an important way of limiting the spread of HIV infection in sub-Saharan Africa. This study also provides the first experimental evidence of MC against HIV infection.

A study was then conducted at the Rakai district in Uganda to investigate the effect of circumcision on the acquisition of HIV by 4996 uncircumcised HIV-negative men [74]. Almost half (2474 individuals) were randomly selected for MC and underwent immediate circumcision while others were delayed circumcision for 24 months [74]. Follow-up information and HIV testing report were recorded for 6, 12, and 24 months. This study shows MC reduced HIV incidence in men without behavioral disinhibition and concluded that circumcision can be recommended for HIV prevention in men [74].

An RCT was also conducted to determine whether MC protects against HIV infection and to assess the safety and changes in sexual behavior related to this intervention [12]. The 2784 young men in Kisumu, Kenya who participated were randomly classified into two groups; the circumcision group (*n* = 1391) and the control group (delayed circumcision groups, 1393) [12]. They were assessed by HIV testing, medical examinations, and behavioral interviews during follow-ups at 1, 3, 6, 12, 18, and 24 months [12]. The finding of this study revealed that MC significantly reduces the risk of HIV acquisition in young men in Africa (Table 1) [12].

### 5.2. Safety, Complications, and Acceptability

One of the major concerns in expanding circumcision services is the safety of the procedure. If appropriate treatment is not provided or left unattended, then the complications of MC can cause excessive pain, excessive bleeding, infection, too much skin removed, anesthetic complications, cosmetic complications, erectile dysfunction, penile damage or amputation, HIV infection, and even death [75]. The chances of complication are high if MC is performed by an inexperienced person under aseptic conditions. Shortage of skilled personnel, shortage of supplies, high cost in private sectors, shortage of dedicated space for counseling, and surgery are some of the limitations for MC.

However, serious complications from MC are rare. The South African, Kenyan, and Ugandan trials reported <4% MC complication rates among the participants. These clinical trials indicate that adult MC can also be safely undertaken even in limited-resource settings if it is performed by well-trained providers [76]. To ensure safe MC, national clinical protocols and quality assurance standards have been developed under the guidelines on the provision of safe circumcision by WHO and UNAIDS [77,78]. As infant circumcision is simpler and safer compared to adult circumcision, WHO and UNAIDS encourage circumcision among infants.

Studies have also suggested that the acceptability rate of circumcision has increased up to 80% in countries like Botswana, Kenya, Malawi, Tanzania, Zambia, South Africa, Uganda, and Zimbabwe [79,80,81]. However, certain factors, like fear of infection and bleeding, pain, and cost, remain barriers to MC. However, resistance to MC persists in some parts of the world, particularly in Asian countries due to cultural practices [76].

### 5.3. Effect of MC on Sexual Function

Studies have been conducted to analyze the effect of MC on sexual functions. RCTs in Kenya show that circumcision does not lead to sexual dysfunction among men while RCTs conducted in Uganda show up to 2% of circumcised men had decreased in sexual satisfaction or sexual dysfunction [82]. Nevertheless, men in the intervention group reported increased penile sensitivity and enhanced ease of reaching orgasm [83,84]. They experienced improved sexual satisfaction and were very satisfied with the outcome of circumcision. The study further suggested that, compared to uncircumcised men, MC has no impact on the frequency of erectile dysfunction, ejaculation, pain during intercourse, lack of pleasure with intercourse, or these dysfunctions combined. Studies from different parts of the world have reported that MC causes no harm and increases sexual satisfaction while a few studies have shown some degree of sexual dysfunction [85,86,87,88] Analysis from systematic review relating to the pros and cons of MC revealed a high quality of evidence that shows MC causes no harm in sexual functions [89].

Studies have also reported on findings from the sexual partner of circumcised males. Studies from Uganda had indicated that women experienced either no change or an increase in sexual satisfaction [90]. The vast majority of women (>95%) in Kenya reported that they were very satisfied with the outcome of circumcision [84]. Studies from other countries, like Malawi and Zambia, have also reported the increased female sexual pleasure with a circumcised partner [91].

### 5.4. Evidence-Based Explanation of MC against the HIV Acquisition

Whether MC prevents men from acquiring HIV during heterosexual intercourse has remained a subject of controversy. Claims have been made that circumcision is a wasteful solution that distracts HIV-infected individuals from using other proven precautionary measures. Thus, MC might result in higher HIV transmission if people start believing it is an alternative precautionary method [50] and start engaging in unprotected sexual activities [50,51,52]. A study was conducted to systematically evaluate the evidence against MC. However, the finding of this study showed that circumcised men are less likely to become infected with HIV [92].

Based on the evidence, various explanations have been made in support of circumcision. The first explanation is that the foreskin of the penis can retain the HIV-1 particles for long periods, and accordingly, continue to infect the glans penis over time; therefore, the removal of the skin covering the tip of the penis may decrease the man’s risk of acquiring HIV infections [58]. Moreover, HIV susceptibility is reduced with the elimination of the subpreputial space as it alters the local immune environment of the penile skin [93,94]. In other words, circumcision reduces the surface area over which HIV-1 infection can take place during heterosexual intercourse, and thus, removal of the foreskin by circumcision reduces the risk for HIV infection.

Another explanation is that circumcision protects HIV-1 acquisition during heterosexual intercourse by reducing the HIV-1 target cells, especially in the inner foreskin surface [21]. Since the inner foreskin mucosa contains a higher mean proportion of HIV-1 target cells with HIV receptors, including CD4 + T-cells, macrophages, and LC [21,34,57,62], it is considered as the point of HIV entry into the penis of an uncircumcised man; therefore, removal of mucosal foreskin epithelium causes a dry keratinized epithelial surface which is more resistant to HIV infection [95].

Furthermore, from an immunological perspective, uncircumcised men are more easily infected by HIV and have higher levels of penile anaerobes compared to circumcised men [96]. This finding further suggests that higher levels of penile anaerobes allow the higher production of immune factors that recruit HIV target cells, such as CD4 + T-cells, to the foreskin. This indicates that anaerobes may modify the risk of HIV infection by triggering inflammation [96]. Moreover, these anaerobes are shared by heterosexual partners and thus associated with increased HIV risk [93,97,98]. For instance, Prodger and Kaul [93], who recently described the immunological basis of how MC reduces HIV susceptibility, observed that immune system activation in foreskin tissues next to subpreputial space promotes HIV infection in uncircumcised men [93]. The role of the genital microbiome was also determined to induce this immune activation [93], and indeed there was a correlation between immune activation and the genital microbiome. These findings indicate that the activation of LC to present HIV-1 to CD4 + T-cells was associated with anoxic surroundings of the subpreputial space. Therefore, in circumcised men, the reduction of anaerobic bacteria protects against HIV infection.

Another study indicated that the uncircumcised penis is more susceptible to trauma, with tearing of the frenulum and thin tissue of the prepuce that is not common in circumcised men [99]. Therefore, the removal of susceptible parts decreases the risk of HIV infection. On an anatomical basis, the benefits of MC against HIV infection have been proven by many investigators based on keratinization of the foreskin [20,21,33,58]. It is now well documented that the keratinization of the inner foreskin is comparatively less than the keratinization in the outer foreskin and because of this reason, uncircumcised men more susceptible to HIV infection [20,33,58]. MC is also strongly supported by the data from the three large RCTs conducted in Africa [11,12,74,100].

## 6. Conclusions

The HIV/AIDS epidemic remains a major disease challenge for the entire world. Nevertheless, in the past four decades, continuous work has led to the development of strategies for HIV/AIDS prevention and management. Despite progress in preventing the spread of HIV in many parts of the world, there are still challenges since protective strategies have failed to control the spread of HIV in many resource-limited settings and high HIV prevalence areas. MC remains an important, evidence-based intervention for the control of generalized HIV epidemics. There is overwhelming immunological evidence in support of MC in preventing the heterosexual acquisition of HIV-1. Moreover, MC is not a new, untested procedure but one of the oldest surgical procedures. It is also worth mentioning that although MC is protective, this protection is indirect and it is not because HIV-1 infection does not occur only over the foreskin, which is removed by circumcision, but also through other tissues of the penis. Nevertheless, considering that there is currently no cure for HIV, and neither is there a cheap, effective, and available preventive vaccine, any level of protection against HIV infection is worthwhile.

## Figures and Tables

**Table 1:** RCTs data showing the efficiency of MC.

Sl.no	RCT Study Site	Year	No. of Participants	Efficiency of MC	Ref
1.	Johannesburg, South Africa	2005	3274	60%	[11]
2.	Rakai district, Uganda	2007	4996	51–60%	[74]
3.	Kisumu, Kenya	2007	2784	53–60%	[12]

## Data Availability

Not applicable.

## References

[1] Cohen M.S., Chen Y.Q., McCauley M., Gamble T., Hosseinipour M.C., Kumarasamy N., Hakim J.G., Kumwenda J., Grinsztejn B., Pilotto J.H. (2011). Prevention of HIV-1 infection with early antiretroviral therapy. N. Engl. J. Med..

[2] Hokello J., Sharma A.L., Dimri M., Tyagi M. (2019). Insights into the HIV Latency and the Role of Cytokines. Pathogens.

[3] Tyagi M., Bukrinsky M. (2012). Human immunodeficiency virus (HIV) latency: The major hurdle in HIV eradication. Mol. Med..

[4] Marzio G., Tyagi M., Gutierrez M.I., Giacca M. (1998). HIV-1 tat transactivator recruits p300 and CREB-binding protein histone acetyltransferases to the viral promoter. Proc. Natl. Acad. Sci. USA.

[5] Sharma A.L., Hokello J., Sonti S., Zicari S., Sun L., Alqatawni A., Bukrinsky M., Simon G., Chauhan A., Daniel R. (2020). CBF-1 Promotes the Establishment and Maintenance of HIV Latency by Recruiting Polycomb Repressive Complexes, PRC1 and PRC2, at HIV LTR. Viruses.

[6] Hokello J., Sharma A.L., Tyagi M. (2020). Efficient Non-Epigenetic Activation of HIV Latency through the T-Cell Receptor Signalosome. Viruses.

[7] Zicari S., Sharma A.L., Sahu G., Dubrovsky L., Sun L., Yue H., Jada T., Ochem A., Simon G., Bukrinsky M. (2020). DNA dependent protein kinase (DNA-PK) enhances HIV transcription by promoting RNA polymerase II activity and recruitment of transcription machinery at HIV LTR. Oncotarget.

[8] Hokello J., Lakhikumar Sharma A., Tyagi M. (2021). AP-1 and NF-kappaB synergize to transcriptionally activate latent HIV upon T-cell receptor activation. FEBS Lett..

[9] Antiretroviral Therapy Cohort C. (2017). Survival of HIV-positive patients starting antiretroviral therapy between 1996 and 2013: A collaborative analysis of cohort studies. Lancet HIV.

[10] Alexaki A., Liu Y., Wigdahl B. (2008). Cellular reservoirs of HIV-1 and their role in viral persistence. Curr. HIV Res..

[11] Auvert B., Taljaard D., Lagarde E., Sobngwi-Tambekou J., Sitta R., Puren A. (2005). Randomized, controlled intervention trial of male circumcision for reduction of HIV infection risk: The ANRS 1265 Trial. PLoS Med..

[12] Bailey R.C., Moses S., Parker C.B., Agot K., Maclean I., Krieger J.N., Williams C.F., Campbell R.T., Ndinya-Achola J.O. (2007). Male circumcision for HIV prevention in young men in Kisumu, Kenya: A randomised controlled trial. Lancet.

[13] Alsallaq R.A., Cash B., Weiss H.A., Longini I.M., Omer S.B., Wawer M.J., Gray R.H., Abu-Raddad L.J. (2009). Quantitative assessment of the role of male circumcision in HIV epidemiology at the population level. Epidemics.

[14] Cook L.S., Koutsky L.A., Holmes K.K. (1994). Circumcision and sexually transmitted diseases. Am. J. Public Health.

[15] Siegfried N., Muller M., Deeks J.J., Volmink J. (2009). Male circumcision for prevention of heterosexual acquisition of HIV in men. Cochrane Database Syst. Rev..

[16] Yuan T., Fitzpatrick T., Ko N.Y., Cai Y., Chen Y., Zhao J., Li L., Xu J., Gu J., Li J. (2019). Circumcision to prevent HIV and other sexually transmitted infections in men who have sex with men: A systematic review and meta-analysis of global data. Lancet Glob. Health.

[17] Boily M.C., Baggaley R.F., Wang L., Masse B., White R.G., Hayes R.J., Alary M. (2009). Heterosexual risk of HIV-1 infection per sexual act: Systematic review and meta-analysis of observational studies. Lancet Infect. Dis..

[18] Jin F., Jansson J., Law M., Prestage G.P., Zablotska I., Imrie J.C., Kippax S.C., Kaldor J.M., Grulich A.E., Wilson D.P. (2010). Per-contact probability of HIV transmission in homosexual men in Sydney in the era of HAART. AIDS.

[19] Szabo R., Short R.V. (2000). How does male circumcision protect against HIV infection?. BMJ.

[20] Morris B.J., Wamai R.G. (2012). Biological basis for the protective effect conferred by male circumcision against HIV infection. Int. J. STD AIDS.

[21] Patterson B.K., Landay A., Siegel J.N., Flener Z., Pessis D., Chaviano A., Bailey R.C. (2002). Susceptibility to human immunodeficiency virus-1 infection of human foreskin and cervical tissue grown in explant culture. Am. J. Pathol..

[22] Cameron D.W., Simonsen J.N., D’Costa L.J., Ronald A.R., Maitha G.M., Gakinya M.N., Cheang M., Ndinya-Achola J.O., Piot P., Brunham R.C. (1989). Female to male transmission of human immunodeficiency virus type 1: Risk factors for seroconversion in men. Lancet.

[23] Hussain L.A., Lehner T. (1995). Comparative investigation of Langerhans’ cells and potential receptors for HIV in oral, genitourinary and rectal epithelia. Immunology.

[24] Sennepin A., Real F., Duvivier M., Ganor Y., Henry S., Damotte D., Revol M., Cristofari S., Bomsel M. (2017). The Human Penis Is a Genuine Immunological Effector Site. Front. Immunol..

[25] Johnson D.C., Huber M.T. (2002). Directed egress of animal viruses promotes cell-to-cell spread. J. Virol..

[26] Igakura T., Stinchcombe J.C., Goon P.K., Taylor G.P., Weber J.N., Griffiths G.M., Tanaka Y., Osame M., Bangham C.R. (2003). Spread of HTLV-I between lymphocytes by virus-induced polarization of the cytoskeleton. Science.

[27] Phillips D.M. (1994). The role of cell-to-cell transmission in HIV infection. AIDS.

[28] Ward A.B., Wilson I.A. (2015). Insights into the trimeric HIV-1 envelope glycoprotein structure. Trends Biochem. Sci..

[29] Dalgleish A.G., Beverley P.C., Clapham P.R., Crawford D.H., Greaves M.F., Weiss R.A. (1984). The CD4 (T4) antigen is an essential component of the receptor for the AIDS retrovirus. Nature.

[30] Deng H., Liu R., Ellmeier W., Choe S., Unutmaz D., Burkhart M., Di Marzio P., Marmon S., Sutton R.E., Hill C.M. (1996). Identification of a major co-receptor for primary isolates of HIV-1. Nature.

[31] Feng Y., Broder C.C., Kennedy P.E., Berger E.A. (1996). HIV-1 entry cofactor: Functional cDNA cloning of a seven-transmembrane, G protein-coupled receptor. Science.

[32] Fischetti L., Barry S.M., Hope T.J., Shattock R.J. (2009). HIV-1 infection of human penile explant tissue and protection by candidate microbicides. AIDS.

[33] McCoombe S.G., Short R.V. (2006). Potential HIV-1 target cells in the human penis. AIDS.

[34] Ganor Y., Zhou Z., Tudor D., Schmitt A., Vacher-Lavenu M.C., Gibault L., Thiounn N., Tomasini J., Wolf J.P., Bomsel M. (2010). Within 1 h, HIV-1 uses viral synapses to enter efficiently the inner, but not outer, foreskin mucosa and engages Langerhans-T cell conjugates. Mucosal. Immunol..

[35] Donoval B.A., Landay A.L., Moses S., Agot K., Ndinya-Achola J.O., Nyagaya E.A., MacLean I., Bailey R.C. (2006). HIV-1 target cells in foreskins of African men with varying histories of sexually transmitted infections. Am. J. Clin. Pathol..

[36] Hirbod T., Bailey R.C., Agot K., Moses S., Ndinya-Achola J., Murugu R., Andersson J., Nilsson J., Broliden K. (2010). Abundant expression of HIV target cells and C-type lectin receptors in the foreskin tissue of young Kenyan men. Am. J. Pathol..

[37] Valladeau J., Ravel O., Dezutter-Dambuyant C., Moore K., Kleijmeer M., Liu Y., Duvert-Frances V., Vincent C., Schmitt D., Davoust J. (2000). Langerin, a novel C-type lectin specific to Langerhans cells, is an endocytic receptor that induces the formation of Birbeck granules. Immunity.

[38] Zhong P., Agosto L.M., Munro J.B., Mothes W. (2013). Cell-to-cell transmission of viruses. Curr. Opin. Virol..

[39] Sewald X., Motamedi N., Mothes W. (2016). Viruses exploit the tissue physiology of the host to spread in vivo. Curr. Opin. Cell Biol..

[40] Chen P., Hubner W., Spinelli M.A., Chen B.K. (2007). Predominant mode of human immunodeficiency virus transfer between T cells is mediated by sustained Env-dependent neutralization-resistant virological synapses. J. Virol..

[41] Jolly C., Kashefi K., Hollinshead M., Sattentau Q.J. (2004). HIV-1 cell to cell transfer across an Env-induced, actin-dependent synapse. J. Exp. Med..

[42] Dimitrov D.S., Broder C.C., Berger E.A., Blumenthal R. (1993). Calcium ions are required for cell fusion mediated by the CD4-human immunodeficiency virus type 1 envelope glycoprotein interaction. J. Virol..

[43] Carr J.M., Hocking H., Li P., Burrell C.J. (1999). Rapid and efficient cell-to-cell transmission of human immunodeficiency virus infection from monocyte-derived macrophages to peripheral blood lymphocytes. Virology.

[44] Martin N., Sattentau Q. (2009). Cell-to-cell HIV-1 spread and its implications for immune evasion. Curr. Opin. HIV AIDS.

[45] Agosto L.M., Uchil P.D., Mothes W. (2015). HIV cell-to-cell transmission: Effects on pathogenesis and antiretroviral therapy. Trends Microbiol..

[46] Del Portillo A., Tripodi J., Najfeld V., Wodarz D., Levy D.N., Chen B.K. (2011). Multiploid inheritance of HIV-1 during cell-to-cell infection. J. Virol..

[47] Russell R.A., Martin N., Mitar I., Jones E., Sattentau Q.J. (2013). Multiple proviral integration events after virological synapse-mediated HIV-1 spread. Virology.

[48] Hubner W., McNerney G.P., Chen P., Dale B.M., Gordon R.E., Chuang F.Y., Li X.D., Asmuth D.M., Huser T., Chen B.K. (2009). Quantitative 3D video microscopy of HIV transfer across T cell virological synapses. Science.

[49] Cameron P.U., Freudenthal P.S., Barker J.M., Gezelter S., Inaba K., Steinman R.M. (1992). Dendritic cells exposed to human immunodeficiency virus type-1 transmit a vigorous cytopathic infection to CD4+ T cells. Science.

[50] Geijtenbeek T.B., Kwon D.S., Torensma R., van Vliet S.J., van Duijnhoven G.C., Middel J., Cornelissen I.L., Nottet H.S., KewalRamani V.N., Littman D.R. (2000). DC-SIGN, a dendritic cell-specific HIV-1-binding protein that enhances trans-infection of T cells. Cell.

[51] Wu L., KewalRamani V.N. (2006). Dendritic-cell interactions with HIV: Infection and viral dissemination. Nat. Rev. Immunol..

[52] Hammonds J.E., Beeman N., Ding L., Takushi S., Francis A.C., Wang J.J., Melikyan G.B., Spearman P. (2017). Siglec-1 initiates formation of the virus-containing compartment and enhances macrophage-to-T cell transmission of HIV-1. PLoS Pathog..

[53] Rappocciolo G., Piazza P., Fuller C.L., Reinhart T.A., Watkins S.C., Rowe D.T., Jais M., Gupta P., Rinaldo C.R. (2006). DC-SIGN on B lymphocytes is required for transmission of HIV-1 to T lymphocytes. PLoS Pathog..

[54] Rappocciolo G., Jais M., Piazza P., Reinhart T.A., Berendam S.J., Garcia-Exposito L., Gupta P., Rinaldo C.R. (2014). Alterations in cholesterol metabolism restrict HIV-1 trans infection in nonprogressors. mBio.

[55] Rinaldo C.R. (2013). HIV-1 Trans Infection of CD4(+) T Cells by Professional Antigen Presenting Cells. Scientifica.

[56] Rappocciolo G., Sluis-Cremer N., Rinaldo C.R. (2019). Efficient HIV-1 Trans Infection of CD4(+) T Cells Occurs in the Presence of Antiretroviral Therapy. Open Forum Infect. Dis..

[57] Ganor Y., Bomsel M. (2011). HIV-1 transmission in the male genital tract. Am. J. Reprod. Immunol..

[58] Dinh M.H., Anderson M.R., McRaven M.D., Cianci G.C., McCoombe S.G., Kelley Z.L., Gioia C.J., Fought A.J., Rademaker A.W., Veazey R.S. (2015). Visualization of HIV-1 interactions with penile and foreskin epithelia: Clues for female-to-male HIV transmission. PLoS Pathog..

[59] Kaushic C. (2011). HIV-1 infection in the female reproductive tract: Role of interactions between HIV-1 and genital epithelial cells. Am. J. Reprod. Immunol..

[60] German Advisory Committee Blood, Subgroup Assessment of Pathogens Transmissible by Blood (S.A.o.P.T.b.B.) (2016). Human Immunodeficiency Virus (HIV). Transfus. Med. Hemother.

[61] Zhou Z., Barry de Longchamps N., Schmitt A., Zerbib M., Vacher-Lavenu M.C., Bomsel M., Ganor Y. (2011). HIV-1 efficient entry in inner foreskin is mediated by elevated CCL5/RANTES that recruits T cells and fuels conjugate formation with Langerhans cells. PLoS Pathog..

[62] Fahrbach K.M., Barry S.M., Anderson M.R., Hope T.J. (2010). Enhanced cellular responses and environmental sampling within inner foreskin explants: Implications for the foreskin’s role in HIV transmission. Mucosal. Immunol..

[63] de Jong M.A., de Witte L., Oudhoff M.J., Gringhuis S.I., Gallay P., Geijtenbeek T.B. (2008). TNF-alpha and TLR agonists increase susceptibility to HIV-1 transmission by human Langerhans cells ex vivo. J. Clin. Investig..

[64] Munch J., Rucker E., Standker L., Adermann K., Goffinet C., Schindler M., Wildum S., Chinnadurai R., Rajan D., Specht A. (2007). Semen-derived amyloid fibrils drastically enhance HIV infection. Cell.

[65] Sabatte J., Ceballos A., Raiden S., Vermeulen M., Nahmod K., Maggini J., Salamone G., Salomon H., Amigorena S., Geffner J. (2007). Human seminal plasma abrogates the capture and transmission of human immunodeficiency virus type 1 to CD4+ T cells mediated by DC-SIGN. J. Virol..

[66] Shaw J.L., Smith C.R., Diamandis E.P. (2007). Proteomic analysis of human cervico-vaginal fluid. J. Proteome. Res..

[67] Wahl S.M., McNeely T.B., Janoff E.N., Shugars D., Worley P., Tucker C., Orenstein J.M. (1997). Secretory leukocyte protease inhibitor (SLPI) in mucosal fluids inhibits HIV-I. Oral Dis..

[68] Spear G.T., Sha B.E., Saarloos M.N., Benson C.A., Rydman R., Massad L.S., Gilmore R., Landay A.L. (1998). Chemokines are present in the genital tract of HIV-seropositive and HIV-seronegative women: Correlation with other immune mediators. J. Acquir. Immun. Defic. Syndr. Hum. Retrovirol..

[69] Padian N.S., Buve A., Balkus J., Serwadda D., Cates W. (2008). Biomedical interventions to prevent HIV infection: Evidence, challenges, and way forward. Lancet.

[70] Galvin S.R., Cohen M.S. (2004). The role of sexually transmitted diseases in HIV transmission. Nat. Rev. Microbiol..

[71] Merz A.J., So M. (2000). Interactions of pathogenic neisseriae with epithelial cell membranes. Annu. Rev. Cell Dev. Biol..

[72] Hogan R.J., Mathews S.A., Mukhopadhyay S., Summersgill J.T., Timms P. (2004). Chlamydial persistence: Beyond the biphasic paradigm. Infect Immun..

[73] Edwards J.L., Apicella M.A. (2004). The molecular mechanisms used by Neisseria gonorrhoeae to initiate infection differ between men and women. Clin. Microbiol. Rev..

[74] Gray R.H., Kigozi G., Serwadda D., Makumbi F., Watya S., Nalugoda F., Kiwanuka N., Moulton L.H., Chaudhary M.A., Chen M.Z. (2007). Male circumcision for HIV prevention in men in Rakai, Uganda: A randomised trial. Lancet.

[75] Muula A.S. (2006). The complications and safety of male circumcision: Implications for HIV prevention. Int. Urol. Nephrol..

[76] Quinn T.C. (2007). Circumcision and HIV transmission. Curr. Opin. Infect. Dis..

[77] WHO, UNAIDS, JHPIEGO (2006). Manual for Male Circumcision under Local Anaesthesia.

[78] WHO (2008). Male Circumcision Quality Assurance: A Guide to Enhancing the Safety and Quality of Services.

[79] Halperin D.T., Fritz K., McFarland W., Woelk G. (2005). Acceptability of adult male circumcision for sexually transmitted disease and HIV prevention in Zimbabwe. Sex. Transm. Dis..

[80] Ngalande R.C., Levy J., Kapondo C.P., Bailey R.C. (2006). Acceptability of male circumcision for prevention of HIV infection in Malawi. AIDS Behav..

[81] Scott B.E., Weiss H.A., Viljoen J.I. (2005). The acceptability of male circumcision as an HIV intervention among a rural Zulu population, Kwazulu-Natal, South Africa. AIDS Care.

[82] Kigozi G., Watya S., Polis C.B., Buwembo D., Kiggundu V., Wawer M.J., Serwadda D., Nalugoda F., Kiwanuka N., Bacon M.C. (2008). The effect of male circumcision on sexual satisfaction and function, results from a randomized trial of male circumcision for human immunodeficiency virus prevention, Rakai, Uganda. BJU Int..

[83] Krieger J.N., Mehta S.D., Bailey R.C., Agot K., Ndinya-Achola J.O., Parker C., Moses S. (2008). Adult male circumcision: Effects on sexual function and sexual satisfaction in Kisumu, Kenya. J. Sex. Med..

[84] Krieger J.N., Bailey R.C., Opeya J.C., Ayieko B.O., Opiyo F.A., Omondi D., Agot K., Parker C., Ndinya-Achola J.O., Moses S. (2007). Adult male circumcision outcomes: Experience in a developing country setting. Urol. Int..

[85] Kim D., Pang M.G. (2007). The effect of male circumcision on sexuality. BJU Int..

[86] Senel F.M., Demirelli M., Misirlioglu F., Sezgin T. (2012). Adult male circumcision performed with plastic clamp technique in Turkey: Results and long-term effects on sexual function. Urol. J..

[87] Waldinger M.D., McIntosh J., Schweitzer D.H. (2009). A five-nation survey to assess the distribution of the intravaginal ejaculatory latency time among the general male population. J. Sex. Med..

[88] Mao L., Templeton D.J., Crawford J., Imrie J., Prestage G.P., Grulich A.E., Donovan B., Kaldor J.M., Kippax S.C. (2008). Does circumcision make a difference to the sexual experience of gay men? Findings from the Health in Men (HIM) cohort. J. Sex. Med..

[89] Friedman B., Khoury J., Petersiel N., Yahalomi T., Paul M., Neuberger A. (2016). Pros and cons of circumcision: An evidence-based overview. Clin. Microbiol. Infect..

[90] Kigozi G., Lukabwe I., Kagaayi J., Wawer M.J., Nantume B., Kigozi G., Nalugoda F., Kiwanuka N., Wabwire-Mangen F., Serwadda D. (2009). Sexual satisfaction of women partners of circumcised men in a randomized trial of male circumcision in Rakai, Uganda. BJU Int..

[91] Shacham E., Godlonton S., Thornton R.L. (2014). Perceptions of Male Circumcision among Married Couples in Rural Malawi. J. Int. Assoc. Provid. AIDS Care.

[92] Westercamp M., Bailey R.C., Bukusi E.A., Montandon M., Kwena Z., Cohen C.R. (2010). Male circumcision in the general population of Kisumu, Kenya: Beliefs about protection, risk behaviors, HIV, and STIs. PLoS ONE.

[93] Prodger J.L., Kaul R. (2017). The biology of how circumcision reduces HIV susceptibility: Broader implications for the prevention field. AIDS Res. Ther..

[94] Hladik F., McElrath M.J. (2008). Setting the stage: Host invasion by HIV. Nat. Rev. Immunol..

[95] Anderson D., Politch J.A., Pudney J. (2011). HIV infection and immune defense of the penis. Am. J. Reprod. Immunol..

[96] Liu C.M., Prodger J.L., Tobian A.A.R., Abraham A.G., Kigozi G., Hungate B.A., Aziz M., Nalugoda F., Sariya S., Serwadda D. (2017). Penile Anaerobic Dysbiosis as a Risk Factor for HIV Infection. mBio.

[97] Schneider J.A., Vadivelu S., Liao C., Kandukuri S.R., Trikamji B.V., Chang E., Antonopoulos D., Prasad S., Lakshmi V. (2012). Increased Likelihood of Bacterial Pathogens in the Coronal Sulcus and Urethra of Uncircumcised Men in a Diverse Group of HIV Infected and Uninfected Patients in India. J. Glob. Infect. Dis..

[98] Liu C.M., Hungate B.A., Tobian A.A., Serwadda D., Ravel J., Lester R., Kigozi G., Aziz M., Galiwango R.M., Nalugoda F. (2013). Male circumcision significantly reduces prevalence and load of genital anaerobic bacteria. mBio.

[99] Alanis M.C., Lucidi R.S. (2004). Neonatal circumcision: A review of the world’s oldest and most controversial operation. Obstet. Gynecol. Surv..

[100] Rasheed Z. (2018). Male circumcision and human immunodeficiency virus infection: An update on randomized controlled trials and molecular evidences. Int. J. Health Sci..

